# Use of a NAT-based assay to improve the surveillance system and prevent transfusion-transmitted malaria in blood banks

**DOI:** 10.1186/s12936-020-03345-y

**Published:** 2020-07-31

**Authors:** Daniele Rocha, Gisely Cardoso de Melo, José Marcelo Hipólito Carneiro, Marisa Ribeiro, Sthefanie Ribeiro, Daniela Tupy de Godoy, Anne Cristine Gomes de Almeida, Elisabete Ferreira de Andrade, Cláudia Maria de Moura Abrahim, Nelson Abrahim Fraiji, Antonio Gomes Pinto Ferreira, Wuelton Marcelo Monteiro, Rodrigo Brindeiro, Amilcar Tanuri, Marcus Vinicius Guimarães de Lacerda, Patrícia Alvarez

**Affiliations:** 1 Instituto de Pesquisa Clínica Carlos Borborema, Fundação de Medicina Tropical Heitor Vieira Dourado, Manaus, Amazonas Brazil; 2 Universidade do Estado do Amazonas, Manaus, Amazonas Brazil; 3 Institute of Technology in Immunobiology Bio-Manguinhos, Oswaldo Cruz Foundation/Fiocruz, Avenida Brasil 4365, Rio de Janeiro, Brazil; 4 Instituto de Pesquisas Leônidas e Maria Deane, Fiocruz, Manaus, Amazonas Brazil; 5 Departamento de Genética, Universidade Federal do Rio de Janeiro-UFRJ, Rio de Janeiro, Brazil; 6 HEMOAM-Fundação Hospitalar de Hematologia e Hemoterapia do Amazonas, Manaus, Amazonas Brazil

**Keywords:** Malaria, *Plasmodium*, Molecular diagnostic, Blood transfusion, Transfusion-transmitted malaria, Hemovigilance, Nucleic acid testing

## Abstract

**Background:**

Malaria can be transmitted by blood transfusion through donations collected from asymptomatic donors. Transfusion-transmitted malaria (TTM) poses a great risk to blood services worldwide. A good screening tool for *Plasmodium* spp. detection in blood banks must have a high sensitivity for prevention of TTM. However, in Brazilian blood banks, screening for malaria still relies on microscopy.

**Methods:**

In Brazil, screening for human immunodeficiency virus type 1 (HIV), RNA/DNA for hepatitis C (HCV) and hepatitis B (HBV) viruses is mandatory for every blood donation and uses nucleic acid amplification testing (NAT). The aim of this study was to evaluate the inclusion of an assay for malaria to identify *Plasmodium* sp. from total nucleic acid (TNA; DNA/RNA) by targeting the *18S rRNA* gene of the parasite.

**Results:**

Considering the limitations of microscopy and the wide availability of the Brazilian NAT platform in the screening of blood units for HIV, HCV, and HBV, a molecular diagnostic tool was validated for detection of *Plasmodium* sp. in blood banks; a pilot study showed that using this novel NAT assay could reduce the risk of TTM.

**Conclusion:**

The prototype HIV/HCV/HBV/malaria NAT assay was effective in detecting infected candidate donors and has good prospects to be applied in routine screening for preventing TTM.

## Background

Malaria is an acute public health problem with annual estimates of 228 million new cases and 405,000 deaths worldwide [1]. *Plasmodium vivax* is the predominant parasite in the Americas, representing 75% of malaria cases [1]. In Brazil, *P. vivax* is also the most common infecting parasite, accounting for 84% of all reported malaria cases [2].

Vector control is the main way to prevent and reduce malaria transmission. Early diagnosis and treatment of malaria reduces disease and prevents deaths and also contributes to reducing malaria transmission [1]. Microscopy is the conventional ‘gold standard’ method for the diagnosis of malaria and allows the differentiation of *Plasmodium* species and the determination of parasite density [3]. Infections not detected by microscopy, often referred to as sub-patent infections, can be detected by molecular methods based on DNA amplification [2]. Compared with microscopy, these methods have demonstrated higher sensitivity, detecting up to 1‒5 parasites/μL of blood, and greater specificity for mixed infections [4]. Identification of *Plasmodium* species by *18S rRNA* gene-targeted molecular assays has been the mainstay of molecular diagnosis of malaria [4, 5].

In addition to vector transmission, malaria can also be transmitted by blood components, and transfusion-transmitted malaria (TTM) cases, although rare, continue to pose a risk to blood services worldwide [6, 7]. TTM is an important public health problem, especially in populations with no immunity to malaria where it can be fatal if not recognized and treated quickly [8]. Infected blood transfusions directly release malaria parasites into a recipient’s bloodstream triggering the development of high-risk complications and potentially leading to a fatal outcome [9, 10]. A systematic review of the epidemiological characteristics of TTM showed that, from 1971 to 2016 in the Americas, there were 63 publications on this subject with 422 cases of TTM, and that no practical, affordable and adequately sensitive screening method is available at blood banks in Latin America, including Brazil [11]. TTM cases were mainly caused by *Plasmodium malariae* (58.4%), *P. vivax* (20.7%), and *Plasmodium falciparum* (17.9%).

Blood transfusion is an essential component of health care, which saves millions of lives across the world. The screening of blood donors for transfusion-transmissible agents is crucial in reducing risks of transfusion of infected units. Appropriate diagnostic tools, tailored to local TTM risk, need to be employed to enhance the safety of the blood supply chain from donors to recipients. In the early 1990s, use of nucleic acid amplification testing (NAT) advanced rapidly, and blood centers started testing blood units using molecular assays for different blood-transmitted infectious diseases [11]. NAT was originally developed for the detection of RNA of human immunodeficiency virus type 1 (HIV) and RNA/DNA of hepatitis C virus (HCV) and hepatitis B virus (HBV) in blood for transfusion [12].

In Brazil, NAT screening for HIV, HCV, and HBV is mandatory for every blood donation. Currently, the Brazilian NAT platform is a national network made available free of charge by the Ministry of Health, screening for these three agents in 100% of the blood donated through the public health system [13]. Due to its relevance in the context of public health, the inclusion of malaria detection into the Brazilian NAT kit is critical for preventing TTM and ensuring transfusion safety, especially in endemic areas in the Amazon basin. Here, a prototype HIV/HCV/HBV/malaria NAT assay was developed and validated. The aim of this study was to evaluate the performance of the Brazilian HIV/HCV/HBV/malaria NAT assay to detect *Plasmodium* sp. from total nucleic acid (TNA; DNA/RNA) by targeting the *18S rRNA* gene; to compare it to the current standard, a validated real-time PCR diagnostic test for malaria targeting the same parasite genomic region; and to incorporate the detection of malaria into a product/platform currently used in the screening of plasma samples in Brazilian public blood banks.

## Methods

### Samples used for validation of the HIV/HCV/HBV/malaria NAT assay

A panel with positive and negative samples was used for the validation of the HIV/HCV/HBV/malaria NAT assay, comprising: (i) 1800 negative samples (whole blood and plasma) from Amazonas Hematology and Hemotherapy Hospital Foundation (HEMOAM); (ii) 16 positive samples (whole blood and plasma) from Dr. Heitor Vieira Dourado Tropical Medicine Foundation (FMT–HVD); and (iii) 31 positive samples from the Malaria Research Laboratory–Oswaldo Cruz Institute (LPM–IOC). Malaria diagnosis was performed by microscopy according to the WHO guidelines [3] and evaluated by an experienced microscopist. Parasite densities were calculated by counting the number of parasites per 500 leukocytes, and the number of parasites/µL per patient was determined [3].

A validated real-time PCR was performed by FMT–HVD to confirm *Plasmodium* sp. infection. FMT–HVD is a national reference center for the treatment of tropical diseases, including malaria. Although an in-house protocol, its real-time PCR is considered the gold standard for the diagnosis of malaria. The extraction of total DNA from whole blood samples was performed using the QIAamp^®^ DNA Blood Mini Kit (Qiagen, Germany) according to the manufacturer’s protocol. All DNA samples were amplified by qMAL Taqman qPCR to detect *Plasmodium* sp. by targeting a conserved region of the *18S rRNA* gene as described elsewhere [14]. DNA was amplified on an Applied Biosystems 7500 Real-Time PCR System (Thermo Scientific, USA). For quantification of *18S rRNA* gene copy numbers, three replicates of plasmid dilutions containing the target region to be quantified (10^2^, 10^4^, 10^6^ copies/μL) were evaluated in each experiment [14].

Samples from the 1st WHO International Standard for *Plasmodium falciparum* DNA for nucleic acid amplification technique (NAT)-based assays (NIBSC code no. 04/176) [15] were also tested during the validation phase in four independent assays at two-fold dilutions ranging from 2.00E+02 to 2.50E+01.

### HIV/HCV/HBV/malaria NAT assay

#### Prototype development and validation

Two different automated nucleic acid isolation workstations were tested to investigate which technology would provide better results in detecting *Plasmodium* sp. gene target using plasma samples. In this phase, BioRobot MDX^®^ (Qiagen) and Chemagic^®^ (PerkinElmer, USA) workstations were used to isolate nucleic acids from FMT–HVD whole blood and plasma samples from 16 patients and LPM plasma samples from 31 patients previously diagnosed with *P. vivax* infection. Of note, both extraction technologies can simultaneously extract DNA and RNA through silica or magnetic beads.

The HIV/HCV/HBV/malaria NAT prototype (Bio-Manguinhos, Brazil) is a real-time nucleic acid amplification multiplex developed to be incorporated into the Brazilian NAT platform to detect HIV, HCV, and HBV [13]. In this new development, the *Plasmodium* sp. detection module was added to the platform together with the HBV detection module. The entire process from sample preparation to detection of amplification was monitored by the inclusion of an internal control (IC) in each individual test. The primers and probes target the C-terminal nucleotide sequence of integrase in HIV (VIC), the 5’UTR of HCV (FAM), and the S region of HBV (FAM) [13]. *Plasmodium18S rRNA* gene was chosen as the malaria target. This well-known parasite genomic region is able to detect all *Plasmodium* species with high sensitivity and has been used elsewhere [14, 16]. To include this target in the Brazilian NAT platform, primers and probes were optimized to be compatible with the multiplex real-time PCR conditions. The *Plasmodium18S rRNA* gene probe was labelled with the Cy5 fluorophore. The prototype HIV/HCV/HBV/malaria NAT assay is run in two replica plates: HIV and HCV are tested in plate 1 and HBV and malaria are tested in plate 2. Amplification reactions were performed under the following conditions: 25 °C for 3 min, 49 °C for 15 min, 85 °C for 7 min, and 50 cycles at 95 °C for 30 s and 60 °C for 40 s. The prototype HIV/HCV/HBV/malaria NAT assay was developed to use plasma as a testing specimen to facilitate the blood screening routine at blood banks. The NAT assay was run in pools of six samples with pooling done as follows: 165 µL of plasma each from six patients plus 10 µL of internal control was mixed for a final volume of 1 mL. The pool was used to isolate nucleic acid that was subsequently used in the assay. If a sample pool tested positive for any molecular target, the individual samples in the pool were tested separately to identify the positive specimen. All samples showing amplification curves with a validated cycle threshold (Ct) < 50 were considered positive for the test.

#### Pilot study

To check the performance and feasibility in a real-life blood bank scenario, the prototype HIV/HCV/HBV/malaria NAT assay (Bio-Manguinhos) was tested on 4745 blood-donor specimens from the HEMOAM blood bank in Manaus, western Brazilian Amazon, using the best nucleic acid extraction/pipetting protocol in parallel with the standard Brazilian NAT platform with the HIV/HCV/HBV NAT kit (Bio-Manguinhos).

## Results

Following development, the prototype HIV/HCV/HBV/malaria NAT assay was challenged with 1800 negative whole blood and plasma samples and a panel of positive blood and/or plasma samples from 47 patients with acute malaria, 16 from FMT–HVD and 31 from LPM, previously diagnosed using microscopy and the validated in-house FMT–HVD real-time PCR protocol. Demographic and clinical characteristics of FMT–HVD samples are described in detail in Table 1. Briefly, 11 patients were men (68.7%), five were women (31.3%), and mean age was 32 years old. The mean asexual parasitaemia was 5811.7 parasites/µL and mean sexual parasite density was 194.5 parasites/µL. All patients were infected with *P. vivax*, 14 (87.5%) samples had rings (mean 2675.5 parasites/µL) and 16 (100%) had trophozoites (mean 3127.1 parasites/µL). In addition, four samples (25%) carried *P. vivax* schizonts (mean 9.1 parasites/µL).

**Table 1:** Demographic and clinical characteristics of FMT–HVD patient samples used for validation of the HIV/HCV/HBV/Malaria NAT assay

Patient	Source	Gender	Age	Parasitaemia (parasites/µL)		Asexual parasitaemia (parasites/µL)		
				Asexual	Sexual	Rings	Trophozoites	Schizonts
1	FMT–HVD	F	26	5757.6	242.4	666.6	5091	0
2	FMT–HVD	M	43	5355.2	644.8	2221	3080.6	53.7
3	FMT–HVD	M	35	6000	0	3060	2940	0
4	FMT–HVD	M	21	6100	0	3468	2632.3	0
5	FMT–HVD	M	37	5861.7	138.3	1078	4783.4	0
6	FMT–HVD	M	20	5847.6	152.4	4476	1352.4	19
7	FMT–HVD	M	35	6000	0	5040	960	0
8	FMT–HVD	F	40	6000	0	0	6000	0
9	FMT–HVD	M	29	5841.7	158.3	1285	4504.4	52.8
10	FMT–HVD	F	32	6000	0	0	6000	0
11	FMT–HVD	M	49	6000	0	4254	1746.5	0
12	FMT–HVD	M	36	4610.7	1389.3	3785	805.4	20.1
13	FMT–HVD	M	17	6000	0	5345	655.2	0
14	FMT–HVD	F	21	6000	0	3558	2441.9	0
15	FMT–HVD	F	34	6000	0	1774	4226.1	0
16	FMT–HVD	M	34	5613.4	386.6	2799	2814.4	0

**Fig. 1:**
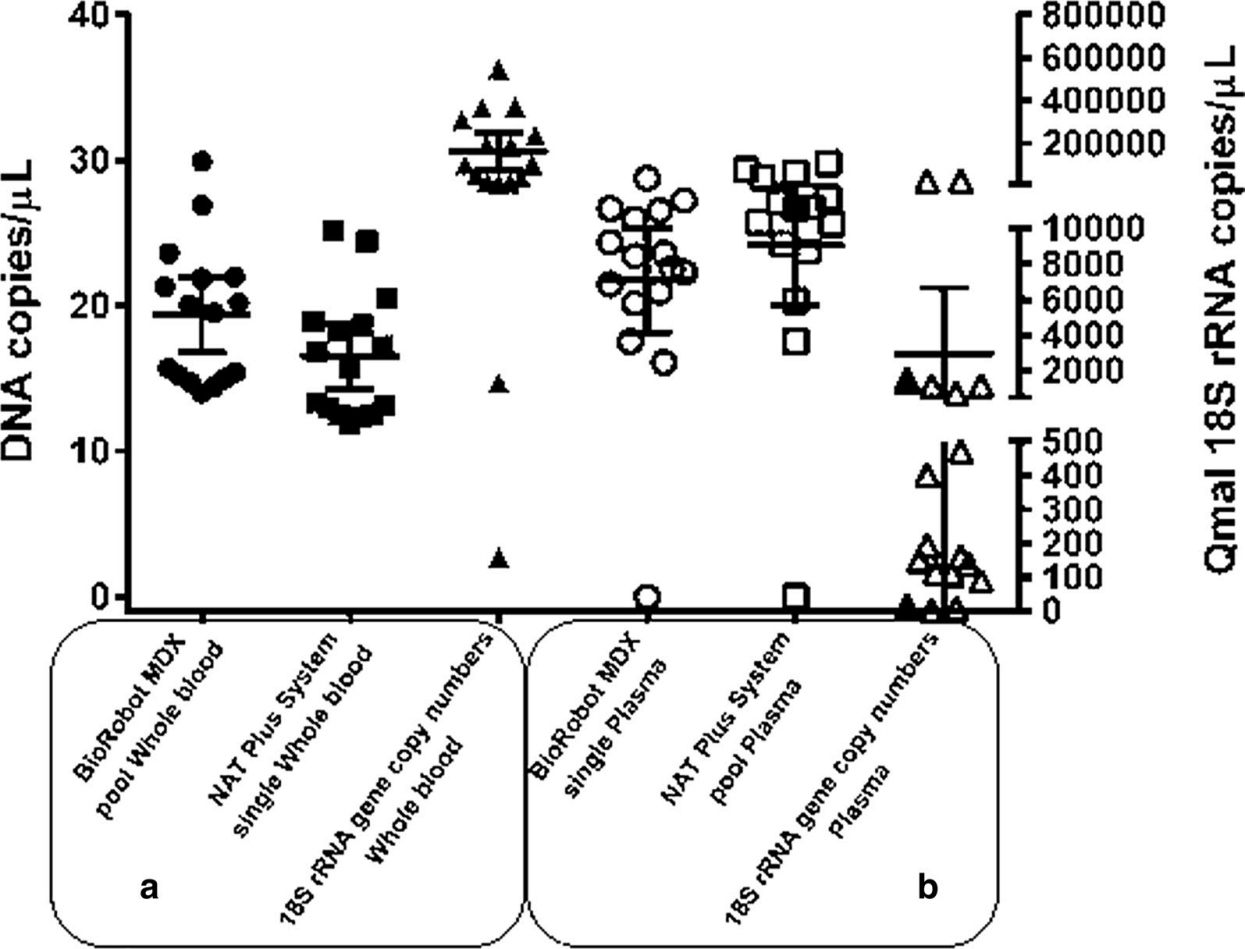
Determination of DNA copies and qMAL *18S rRNA* copies of *Plasmodium vivax* in samples from blood donors from Manaus, western Amazon, Brazil. **a** Determination of DNA copies in pooled whole blood samples (BioRobot MDx, silica) and single whole blood samples (NAT Plus, beads), and qMAL *18S rRNA* gene copy numbers in whole blood samples. **b** Determination of DNA copies in single plasma samples (BioRobot MDx, silica) and pooled plasma samples (NAT Plus System, beads), and qMAL *18S rRNA* gene copy numbers in plasma samples

Fourteen (87.5%) pooled plasma samples and 15 single plasma samples (93.8%) from the FMT–HVD tested with the BioRobot MDX workstation were positive to *Plasmodium* sp. (Table 2). Of note, 100% of the whole blood positive samples tested with the BioRobot MDX system were positive to *Plasmodium* sp. In addition, all (100%) positive plasma samples (single and pooled) were detected when tested using the Chemagic workstation, hereafter named NAT Plus System (Table 2), showing 100% concordance with microscopy (Fig. 1). Similarly, all (100%) LPM single and pooled samples were detected when using the NAT Plus System, showing 100% concordance with microscopy. The Ct values from the individual testing are summarized in Table 3.

**Table 2:** Cycle threshold (Ct) results for FMT–HVD samples using the BioRobot MDx (silica extraction) and NAT Plus System (bead extraction) workstations, and in-house *18S RNA* gene quantification

Patient	Sample	MDx (silica)		NAT Plus (beads)		*18S RNA* quantification
		Single (Ct value)	Pool of six (Ct value)	Single (Ct value)	Pool of six (Ct value)	Copies/µL
1	Whole blood	12.45	14.36			550,345.9
	Plasma	16.1	17.53	15.64	18.51	22,732.9
2	Whole blood	18.66	20.22			38,766.9
	Plasma	17.53	20.33	21.41	19.91	89.5
3	Whole blood	20.47	23.61			13,727.3
	Plasma	25.99	28.83	20.01	24.96	150.8
4	Whole blood	13.17	15.17			53,443.5
	Plasma	23.42	27.4	19.58	22.95	161.7
5	Whole blood	17.19	21.87			194,645.8
	Plasma	20.95	24.23	17.45	21.08	399.9
6	Whole blood	15.7	21.29			103,623.2
	Plasma	20.16	26.7	15.39	19.74	192.9
7	Whole blood	18.2	20.08			368,428.4
	Plasma	22.32	25.61	19.75	23.38	24,047.6
8	Whole blood	25.22	29.92			161.5
	Plasma	Non-reagent	Non-reagent	28.12	31.84	7.1
9	Whole blood	18.91	21.97			14,431.3
	Plasma	23.61	26.75	20.51	24.09	1155.3
10	Whole blood	24.43	26.93			1358.9
	Plasma	28.76	Non-reagent	26.07	28.49	2.9
11	Whole blood	12.35	14.71			186,777.7
	Plasma	26.53	29.1	22.52	25.74	471.7
12	Whole blood	11.89	14.0			239,674.9
	Plasma	26.7	29.36	22.67	25.9	109.2
13	Whole blood	13.37	15.71			372,567.5
	Plasma	22.57	25.73	18.3	21.44	1156.3
14	Whole blood	16.86	19.5			19,880.9
	Plasma	27.23	29.8	21.84	25.84	113.2
15	Whole blood	12.53	14.96			310,122.1
	Plasma	24.38	27.1	19.79	23.64	137.9
16	Whole blood	12.94	15.39			102,256.4
	Plasma	21.48	23.74	16.23	19.86	739.8

**Table 3:** Cycle threshold (Ct) values for LPM–IOC samples tested individually with the NAT Plus System

Patient	Source	Gender	Microscopy/real-time PCR	NAT plus (Ct value)
1	LPM–IOC	M	*P. vivax*	29.86
2	LPM–IOC	M	*P. vivax*	30.76
3	LPM–IOC	F	*P. vivax*	32.34
4	LPM–IOC	M	*P. vivax*	32.42
5	LPM–IOC	M	*P. vivax*	29.71
6	LPM–IOC	M	*P. vivax*	31.4
7	LPM–IOC	F	*P. vivax*	30.07
8	LPM–IOC	M	*P. vivax*	32.61
9	LPM–IOC	M	*P. vivax*	31.76
10	LPM–IOC	M	*P. vivax*	31.35
11	LPM–IOC	M	*P. vivax*	33.42
12	LPM–IOC	M	*P. vivax*	28.23
13	LPM–IOC	M	*P. vivax*	32.78
14	LPM–IOC	M	*P. vivax*	33.6
15	LPM–IOC	M	*P. vivax*	31.3
16	LPM–IOC	M	*P. vivax*	26.42
17	LPM–IOC	F	*P. vivax*	26.62
18	LPM–IOC	M	*P. vivax*	32.5
19	LPM–IOC	M	*P. vivax*	29.5
20	LPM–IOC	F	*P. vivax*	30.84
21	LPM–IOC	F	*P. vivax*	26.32
22	LPM–IOC	F	*P. vivax*	29.35
23	LPM–IOC	M	*P. vivax*	32.17
24	LPM–IOC	M	*P. vivax*	30.82
25	LPM–IOC	M	*P. vivax*	35.9
26	LPM–IOC	M	*P. vivax*	30.83
27	LPM–IOC	M	*P. vivax*	31.27
28	LPM–IOC	M	*P. vivax*	27.78
29	LPM–IOC	M	*P. vivax*	31.48
30	LPM–IOC	M	*P. vivax*	32.85
31	LPM–IOC	M	*P. vivax*	29.73

To check the specificity of the prototype NAT assay, 1800 truly negative samples were tested and no false positive signal was identified in the malaria amplification module. This finding confirms the high specificity of the prototype HIV/HCV/HBV/malaria NAT assay for the malaria target. The analysis of 47 truly positive samples and 1800 truly negative samples revealed a specificity of 99.8‒100% [95% confidence interval (CI)], a sensitivity of 92.45‒100% (95% CI), and an accuracy of 99.8‒100% (95% CI).

The prototype HIV/HCV/HBV/malaria NAT assay detected all dilutions tested using the reference material from the 1st WHO International Standard for *Plasmodium falciparum* DNA nucleic acid amplification technique (NAT)-based assays (NIBSC code no. 04/176) [15]. Eight replicates were tested at each dilution and only one replicate was not detected at the lower dilution (Table 4).

**Table 4:** Cycle threshold (Ct) values for the 1st WHO International Standard samples tested using the HIV/HCV/HBV/malaria NAT assay (Bio-Manguinhos)

Replicate	Concentration tested			
	2.00E02 (Ct value)	1.00E02 (Ct value)	5.00E01 (Ct value)	2.50E01 (Ct value)
1	32.56	33.66	33.79	35.75
2	32.63	32.94	36.24	43.99
3	32.39	32.88	37.39	34.23
4	31.92	32.76	34.42	47.63
5	32.39	33.31	34.17	34.96
6	32.05	33.73	34.48	37.67
7	32.73	33.17	33.23	ND
8	33.1	33.31	33.64	35.39
Mean (SD)	32.47 (± 0.38)	33.22 (± 0.35)	34.67 (± 1.42)	38.52 (± 5.2)

*ND* not detected, *SD* standard deviation

Results of the pilot study in a real-life blood bank scenario with specimens from 4745 donors are summarized in Table 5. The results for HIV, HCV, and HBV were concordant between the standard HIV/HCV/HBV NAT assay and the prototype HIV/HCV/HBV/malaria NAT assay. Of the 4745 plasma samples tested, three donors showed a positive signal on PCR to the malaria probe (prevalence of 0.06%, 95% CI 0.02 to 0.18%) and their blood units were discarded. These positive samples were further confirmed by the validated real-time PCR performed at FMT–HVD, and parasitaemia in these samples ranged from 13 to 1410 copies/µL. The three donors were men with a mean age of 33 years and Ct values in these three specimens ranged from 24.07 to 29.09 for pooled samples and 20.01 to 26.54 for single samples. Additionally, the donors were recalled for repeat blood collection and confirmation of the diagnosis by gold standard microscopy.

**Table 5:** Cycle threshold (Ct) values and *18S RNA* gene quantification in malaria-positive plasma samples from the pilot study at HEMOAM, Manaus, Brazil

Patient	Source	Gender	Age	NAT Plus (beads)		*18S RNA* quantification
				Single (Ct value)	Pool of six (Ct value)	Copies/µL
17	HEMOAM	M	46	26.16	NT^a^	NT^a^
18	HEMOAM	M	24	26.54	29.09	13.01
19	HEMOAM	M	29	20.01	24.07	1454.0

*NT* not tested

^a^There was not enough material to perform all tests

## Discussion

Although advanced testing techniques are now becoming available and are being adopted at many centers, the risk of contracting transfusion-transmitted infections after transfusion of blood or blood components persists. Malaria transmitted by blood transfusion has been described since the early 1900s. The risk of transmitting malaria by blood transfusion in countries where malaria transmission is endemic can be high, and healthcare systems need alternatives for detecting the parasite or its components in the blood of potentially infected donors [17, 18].

Haemovigilance in Brazil regarding malaria differs between malaria-endemic and non-endemic regions. Blood units must be tested for the presence of *Plasmodium* sp. or plasmodial antigens in endemic areas, whereas in non-endemic areas candidate donors are rejected if they have travelled to endemic areas in the previous 3 months or had malaria in the previous 3 years, posing difficulty in finding suitable donors in the Amazon region. The Brazilian Amazon has large cities with millions of malaria-naïve individuals susceptible to infection. In these regions, a better tool for diagnosing malaria is needed and it is currently challenging to find a technique for screening blood donors that is easy to perform and can screen a large number of samples with high sensitivity [19]. PCR has been shown to be an effective tool for diagnosing human malaria parasites, even in mixed infections, and has proven to be a superior method to microscopy [20, 21].

The Brazilian NAT platform was a milestone in screening infectious diseases in Brazil and increased the safety of transfused blood [13]. The platform was born from a demand by the Ministry of Health to improve the safety of haematologic and haemotherapy health care. The Brazilian NAT platform, entirely developed in Brazil and including a continuous improvement programme, has identified the need for a more modern platform with magnetic bead extraction and the addition of a malaria target [13]. This study presents the validation results of the prototype HIV/HCV/HBV/malaria NAT assay to be used in the Brazilian NAT platform for screening blood donated through the public health system in Brazil.

Results for the two molecular biology workstations tested, BioRobot MDX (silica technology) and NAT Plus System (bead technology), showed that the latter was able to detect TNA from *Plasmodium* sp. in plasma samples, both in six-pooled and single sample testing. Additionally, the volume of blood analysed can affect the sensitivity of the assay; the BioRobot MDX uses 285 µL of plasma sample for TNA isolation, whereas the NAT Plus System uses 1000 µL, which has enabled the incorporation of the malaria target into a product/platform currently in use in Brazilian public blood banks in the screening of plasma samples. Currently, 100% of the blood donated through public blood banks is screened by the Brazilian NAT platform for HIV, HCV, and HBV [13] and the incorporation of the malaria target into this platform is a great advance in the safety of blood donation in Brazil.

The validation results of the Brazilian prototype HIV/HCV/HBV/malaria NAT assay revealed a specificity of 99.8‒100% (CI 95%), a sensitivity of 92.45‒100% (CI 95%), and an accuracy of 99.8‒100% (CI 95%). No false-positives were detected when testing 1800 truly negative samples. The sensitivity of the assay, according to the Technical Dossier for In Vitro Diagnostic Products submitted to the Brazilian regulatory agency ANVISA, is 200 copies/mL with a limit of detection (LoD) of 60.97 copies/mL (95% CI) (unpublished data). Of note, the HIV/HCV/HBV/malaria NAT assay was able to detect replicates even at concentrations below the LoD. However, as concentrations are reduced, standard deviation increases leading to failure to detect replicates.

In the pilot study with 4745 blood donor specimens from standard HEMOAM routine, the prototype HIV/HCV/HBV/malaria NAT assay detected three donors infected with *Plasmodium* sp. These donors were deemed fit for donation following the predonation interview and the NAT assay was able to detect TNA of *Plasmodium* in their plasma samples.

A systematic review in Brazil reported variable malaria prevalence by PCR in candidate blood donors from different geographic origins, including laboratory-tested blood donors, deferred donors, and blood donors suspected to be responsible for TTM cases. The prevalence of TTM was 0.3% in Manaus (n = 286), 1% in Rio Branco (n = 100), 1.3% in the state of Pará (n = 595), 2% in Belém (n = 100), 3% in Macapá (n = 100) and Porto Velho (n = 100), and 7.5% in São Paulo (n = 1108) [22]. TTM prevalence in the current study was much lower (0.06%) than previously reported and the difference between studies is likely due to the type of sample tested. The current study used only samples deemed suitable for donation, whereas other studies used samples from deferred donors. The frequency of TTM has been estimated at one case per four million units donated in non-endemic areas [22].

Only samples infected with *P. vivax* were detected in this pilot study. This result can be explained by the fact that in the study area, *P. vivax* causes approximately 85% of malaria episodes [22]. The *18S rRNA* gene target selected for malaria detection was a logical choice given its high sequence conservation and the fact that each *Plasmodium* parasite is known to harbor five copies of the *18S rRNA* gene [16].

A screening tool for detecting malaria infection in Brazilian blood banks, both in endemic and non-endemic regions, must have high sensitivity and specificity. Considering the limitations of microscopy and the wide availability of the Brazilian NAT platform in the screening of blood units for HIV, HCV, and HBV, this study validated a molecular diagnostic tool for detection of *Plasmodium* sp. in blood banks, reducing the risk of TTM.

## Conclusions

The prototype HIV/HCV/HBV/malaria NAT assay used in the Brazilian NAT Plus platform is a promising alternative for screening malaria in blood banks of endemic and non-endemic regions, with good analytic sensitivity and accurate methodology to detect malaria parasites. The HIV/HCV/HBV/malaria NAT assay is an easy and fast screening method that takes advantage of a platform currently in use as part of the blood bank routine and which has been effective for improving blood transfusion safety.

## Data Availability

Please contact corresponding author for additional data requests.

## Abbreviations

TTM: Transfusion-transmitted malaria
TNA: Total nucleic acid
NAT: Nucleic acid amplification testing
FMT–HVD Dr.: Heitor Vieira Dourado Tropical Medicine Foundation
LPM–IOC: Malaria Research Laboratory–Oswaldo Cruz Institute
WHO: World Health Organization
HEMOAM: Amazonas Hematology and Hemotherapy Hospital Foundation
IC: Internal control
Ct: Cycle threshold
HIV: Human immunodeficiency virus
HCV: Hepatitis C Virus
HBV: Hepatitis B Virus
