# Correction to: Use of a NAT-based assay to improve the surveillance system and prevent transfusion-transmitted malaria in blood banks

**DOI:** 10.1186/s12936-020-03396-1

**Published:** 2020-09-04

**Authors:** Daniele Rocha, Gisely Cardoso de Melo, José Marcelo Hipólito Carneiro, Marisa Ribeiro, Sthefanie Ribeiro, Daniela Tupy de Godoy, Elaine Costa, Anne Cristine Gomes de Almeida, Elisabete Ferreira de Andrade, Cláudia Maria de Moura Abrahim, Nelson Abrahim Fraiji, Antonio Gomes Pinto Ferreira, Wuelton Marcelo Monteiro, Rodrigo Brindeiro, Amilcar Tanuri, Marcus Vinicius Guimarães de Lacerda, Patrícia Alvarez

**Affiliations:** 1 Instituto de Pesquisa Clínica Carlos Borborema, Fundação de Medicina Tropical Heitor Vieira Dourado, Manaus, Amazonas Brazil; 2 Universidade do Estado do Amazonas, Manaus, Amazonas Brazil; 3 Institute of Technology in Immunobiology Bio-Manguinhos, Oswaldo Cruz Foundation/Fiocruz, Avenida Brasil, 4365 Rio de Janeiro Brazil; 4 Instituto de Pesquisas Leônidas e Maria Deane, Fiocruz, Manaus, Amazonas Brazil; 5 Departamento de Genética, Universidade Federal do Rio de Janeiro-UFRJ, Rio de Janeiro, Brazil; 6 HEMOAM-Fundação Hospitalar de Hematologia e Hemoterapia do Amazonas, Manaus, Amazonas Brazil

## Correction to: Malar J (2020) 19:275 10.1186/s12936-020-03345-y

Following publication of the original article [1], the authors flagged that an author has been erroneously excluded from the article’s authorship.

The missing author is Elaine Costa, who was part of the team that validated the NAT platform, did the pilot study and analyzed the results.

To correct this error, please find Elaine Costa included in the author list of this correction.

In addition, please see here for the (corrected) Author Contributions statement:

GCM, ACGA, JHC, CMMA, NAF were responsible for patient recruitment and laboratory procedures; DR, MR, SR,EC, EFA validated the NAT platform and did the pilot study; DR, GCM, DTG, AT analyzed the results and wrote first draft of the manuscript; PA, GCM, AGPF, AT, WMM, and MVGL analyzed the results, revised and approved the final version of the manuscript.

The authors apologize for any inconvenience caused.
